# Kerion in Disguise: Avoiding Surgical Misdiagnosis in Adolescent Scalp Lesions

**DOI:** 10.1002/ccr3.72827

**Published:** 2026-05-29

**Authors:** Aliza Paudyal, Parbatraj Regmi, Sudha Agrawal

**Affiliations:** ^1^ Department of Dermatology and Venerology B. P. Koirala Institute of Health Sciences Dharan Nepal; ^2^ Department of Surgery B. P. Koirala Institute of Health Sciences Dharan Nepal

**Keywords:** adolescent, kerion, scarring alopecia, surgical mimic, tinea capitis

## Abstract

Kerion can mimic a surgical condition, and its early recognition is critical to avoid unnecessary procedures, antibiotics, and financial burden.

## 1 Introduction

Tinea capitis (TC) is a superficial dermatophytic infection that involves hair follicles and the scalp, presenting with diverse clinical manifestations. These may be alopecia with or without broken hairs, localized or diffuse scaling of variable intensity, scaling, pustules, crusting, and erythema. The presentation may resemble other inflammatory or infectious scalp disorders, such as impetigo, folliculitis declavans, abscess, or cellulitis [1]. The dermatophytes most commonly isolated in adult cases are Trichophyton tonsurans, Trichophyton violaceum, Microsporum canis, etc. [2]. Clinically typical kerion type of TC is rare; in adults, it shows a polymorphic atypical clinical presentation, which is difficult to diagnose, and it will subsequently delay treatment [3]. Herein, we present a case of kerion in a young adult misdiagnosed as a skin and soft tissue infection of the scalp.

## 2 Case History/ Examination

**FIGURE 1:**
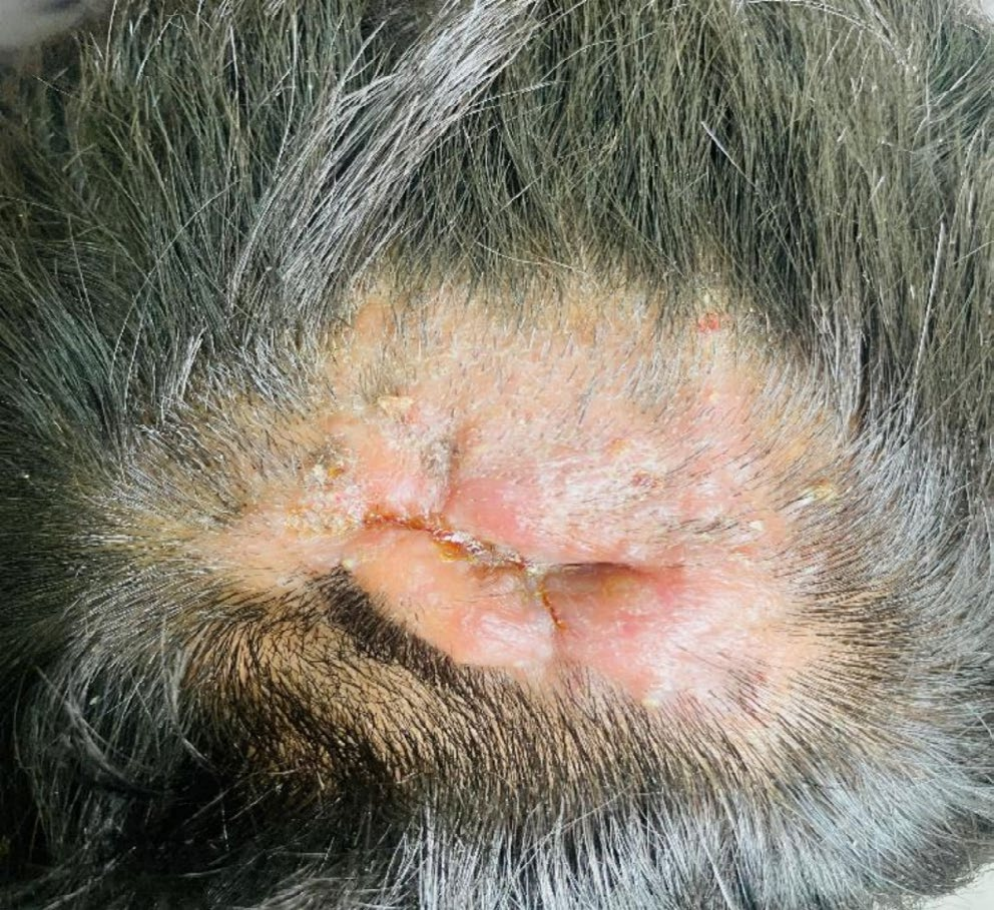
This picture shows an erythematous plaque on the scalp with central ulceration, yellowish crusting, scaling, alopecia, and linear indenting scarring.

A 17‐year‐old adolescent male presented with a one‐month history of a hairless, desquamating, and swollen lesion on the occipital scalp. He initially visited the surgery department, where incision and drainage (I&D) was performed, and systemic antibiotic flucoxallin 500 mg qid and cefuroxime 500 mg BD were prescribed for 2 weeks. Still, the lesion did not improve on treatment. The patient was referred to the dermatology department. Dermatology referral revealed an irregular erythematous plaque on the scalp with central ulceration following incision and drainage, yellow crusting, scaling, alopecia, and linear indenting scarring (Figure 1). The patient did not give a history of any animal contacts.

## 3 Differential Diagnosis, Investigations, and Treatment

**FIGURE 2:**
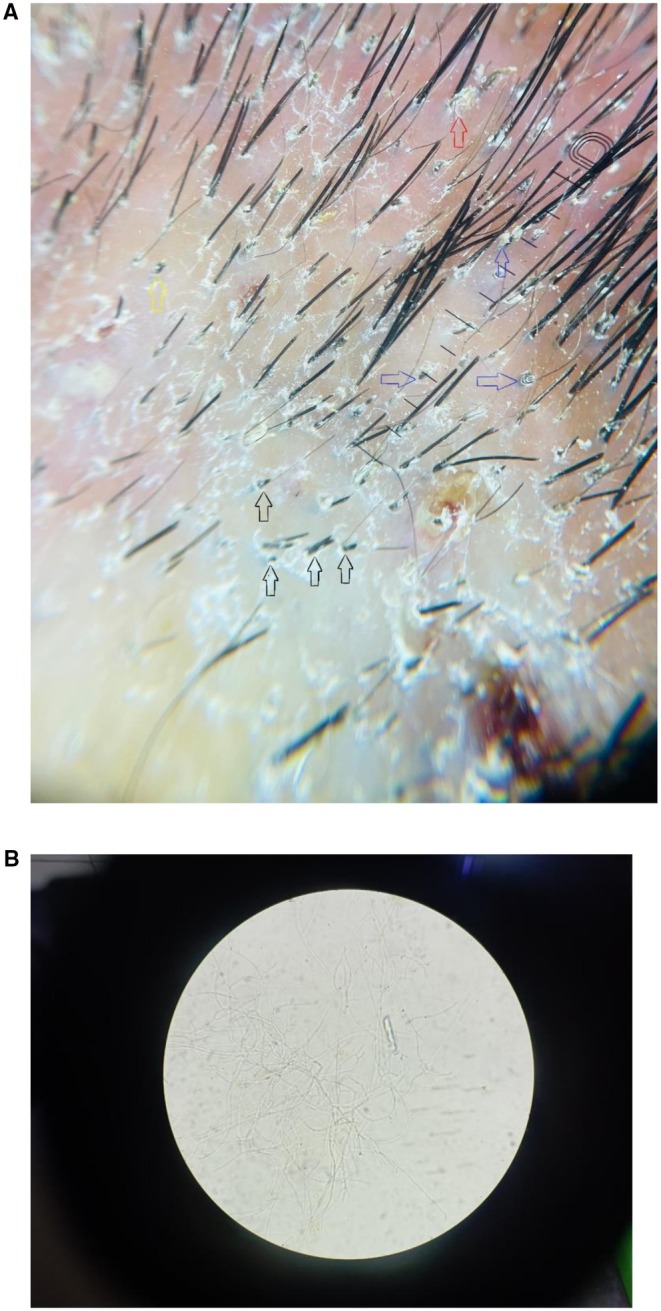
(A) Dermoscopic features of Tinea Capitis. Black arrowhead: Different sizes broken hair; blue arrowhead: Black dot; yellow arrowhead: Comma hair; red arrowhead: Perifollicular scaling. (B) KOH image showing fungal elements (10×).

Dermoscopy demonstrated comma hairs, black dots, multiple sizes of broken hair, black dot, and perifollicular scaling (Figure 2A). Potassium hydroxide (KOH) preparation of scalp scrapings confirmed the presence of fungal elements suggestive of dermatophytes (Figure 2B), establishing the diagnosis of Tinea capitis. Complete blood count, serology, and fungal culture were performed, which came out to be negative. The patient denied participation in contact or close‐contact sports, including wrestling, judo, or soccer. He also denied taking part in school or club activities involving frequent skin‐to‐skin contact. There was no history of sharing personal items such as helmets, combs, or towels, and he did not live in a dormitory or hostel. In addition, the patient denied any recent barbershop or hairdresser visits. The patient denied any systemic symptoms, and his lymph node was non‐palpable. Histopathology and wood's lamp were not performed in our case. The patient was treated with oral griseofulvin (ultramicrosize) 500 mg in the morning and 250 mg in the evening, and topical clotrimazole twice daily, antihistamine cetirizine 10 mg OD in the evening and along with ketoconazole 2% shampoo on alternate days.

## 4 Conclusion and Results (Outcome and Follow‐Up)

**FIGURE 3:**
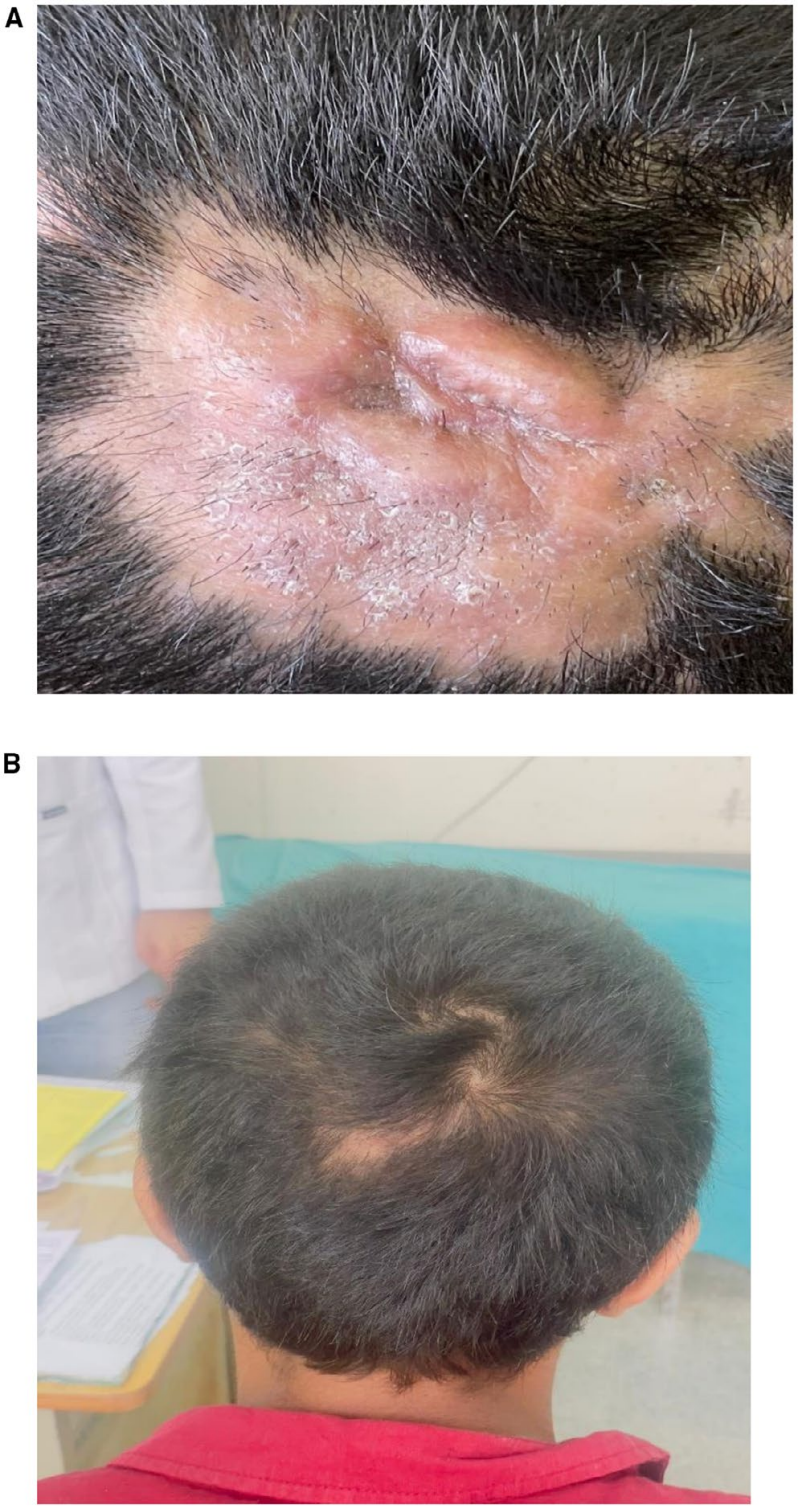
(A) (4 week follow‐up): Short broken hairs, whitish scaling. (B) (12‐week follow‐up): Hair growth seen with minimal residual alopecia.

Patient had complete resolution within 4 weeks (Figure 3A) and hair regrowth at 8 weeks, with minimal residual scarring (Figure 3B). This case of kerion highlights the diagnostic challenge in adolescents, underscores the importance of considering fungal infections in atypical scalp lesions, and reinforces the value of accurate diagnosis and timely antifungal therapy to prevent unnecessary surgical interventions, local anesthesia, scarring alopecia, morbidity, and financial burden.

## 5 Discussion

Kerion is observed more frequently in low socioeconomic conditions, inadequate hygiene, predominantly affecting children aged between 3 and 7 years, with a higher incidence in males. The pathogenesis is primarily attributed to vigorous, hypersensitivity‐mediated immune responses orchestrated by T‐cell lymphocytes [4, 5]. Kerion is typically characterized by boggy swelling, purulent discharge, alopecia, and lymph node enlargement, which can lead physicians to confuse it with pyoderma, especially an abscess [6, 7]. Bogginess of TC sometimes could be a nightmare for surgeons as it can mimic the most common conditions like bacterial pyoderma, scalp cellulitis, or folliculitis decalvans. A severely inflammatory kerion can cause dermal fibrosis and follicular destruction, ultimately resulting in scarring alopecia [8].

TC in adults is about 11%–13% in Asia, and females are frequently affected, especially post‐menopausal women [9]. Wood's lamp, dermoscopy, and potassium hydroxide (KOH) mount are easy and convenient for the diagnosis of TC, whereas fungal culture can come back negative in most cases, and histopathology is rarely performed [10]. The selection of an oral antifungal agent is guided by patients' characteristics, oral griseofulvin, terbinafine, or itraconazole, and treatment should be continued for a minimum of 6–8 weeks [11]. Dermoscopy of TC shows comma hairs, corkscrew hairs, Morse code‐like hairs, zigzag hairs, bent hairs, block hairs, and i‐hairs. Other common, but not characteristic, dermoscopic features were broken hairs, black dots, perifollicular scaling, and diffuse scaling [12].

Our patient's scalp lesions were initially misdiagnosed as scalp abscesses, and incision and drainage were performed along with oral antibiotics by a surgeon. With no improvement, the patient visited the dermatology department, where clinical lesions, KOH mount, and dermoscopy aided in the diagnosis of kerion. He responded well to oral griseofulvin (ultramicrosize) 10 mg/kg/day along with topical cotrimazole, achieving resolution of plaque and approximately 95% hair regrowth in the affected area, with minimal residual scarring alopecia noted. The presence of a purulent scalp lesion with accompanying hair loss should prompt consideration of tinea capitis or kerion and may be more consistent with a fungal infection than a bacterial abscess.

## Author Contributions

**Aliza Paudyal:** conceptualization, data curation, investigation, methodology, writing – review and editing. **Parbatraj Regmi:** writing – review and editing. **Sudha Agrawal:** supervision, validation.

## Funding

The authors have nothing to report.

## Ethics Statement

Ethical clearance has been obtained from the Institutional Review Committee.

## Consent

The patient in this manuscript has given written informed consent for the publication of case details.

## Conflicts of Interest

The authors declare no conflicts of interest.

## Data Availability

The materials used in this study are available from the corresponding authors upon request.
